# Cip29 is phosphorylated following activation of the DNA damage response in *Xenopus* egg extracts

**DOI:** 10.1371/journal.pone.0181131

**Published:** 2017-07-17

**Authors:** Janet Holden, Elaine M. Taylor, Howard D. Lindsay

**Affiliations:** Lancaster Medical School, Faculty of Health and Medicine, Lancaster University, Lancaster, United Kingdom; Saint George's University, UNITED KINGDOM

## Abstract

Acting through a complex signalling network, DNA lesions trigger a range of cellular responses including DNA repair, cell cycle arrest, altered gene expression and cell death, which help to limit the mutagenic effects of such DNA damage. RNA processing factors are increasingly being recognised as important targets of DNA damage signalling, with roles in the regulation of gene expression and also more directly in the promotion of DNA repair. In this study, we have used a X*enopus laevis* egg extract system to analyse the DNA damage-dependent phosphorylation of a putative RNA export factor, Cip29. We have found that Cip29 is rapidly phosphorylated in response to DNA double-strand breaks in this experimental system. We show that the DNA damage-inducible modification of Cip29 is dependent on the activity of the key double-strand break response kinase, ATM, and we have identified a conserved serine residue as a damage-dependent phosphorylation site. Finally, we have determined that Cip29 is not required for efficient DNA end-joining in egg extracts. Taken together, these data identify Cip29 as a novel target of the DNA damage response and suggest that the damage-dependent modification of Cip29 may relate to a role in the regulation of gene expression after DNA damage.

## Introduction

A hallmark of cancer, genome instability is recognised as a key driving force in cancer progression, since it facilitates the acquisition of further tumourigenic changes [1]. Genome instability may result from exposure to DNA damaging agents, exogenous or endogenous in origin, or as a consequence of the replicative stress which generally accompanies oncogenic transformation [2, 3]. A variety of DNA damage response (DDR) pathways exist to contend with such genetic damage. Principal amongst these is DNA damage signalling via the phosphatidylinositol 3-kinase-like kinases (PI3KK), ATM (ataxia telangiectasia mutated) and ATR (ataxia telangiectasia and Rad3 related), which elicits a variety of cellular responses, including activation of DNA repair mechanisms, cell cycle arrest or apoptosis [4]. These outcomes are achieved through phosphorylation-dependent modulation of protein function and also as a result of altered gene expression, by activation of DNA damage-inducible transcription, and through post-transcriptional changes in mRNA processing and translation. Of these, RNA processing has only relatively recently started to be recognised as an important element of the DDR [5, 6].

Transcription is tightly coupled with a series of RNA processing events in which the primary transcript undergoes 5’-end capping, splicing, 3’-end processing and nuclear export [7]. DNA damage-dependent regulation of gene expression is, at least in part, achieved through alterations in RNA processing. DNA damage represses 3’-end processing, alters the pattern of pre-mRNA splicing and decreases mRNA stability, leading to a general reduction in gene expression, while mRNAs encoding DDR proteins may selectively escape this repression through the binding of specific RNA binding proteins (RBPs) [8–13]. As well as regulating gene expression in response to DNA damage, RNA processing plays an important role in averting genome instability by preventing formation of DNA/RNA hybrids (R-loops) during transcription [14]. R-loops are formed when the nascent RNA anneals to the DNA template strand, leaving the non-transcribed DNA strand unpaired and susceptible to breakage. Protein complexes involved in transcription elongation and mRNA processing and export, along with the BRCA2 tumour suppressor, have been shown to prevent deleterious R-loop formation [15, 16]. Moreover, several RNA processing factors are known to localise to sites of DNA damage and to interact with DNA repair proteins, suggesting that they may play a direct role in the DDR [17–21]. It is perhaps unsurprising then, that several proteomic and functional screens have identified RNA processing factors as targets of DNA damage signalling or as participants in the DDR [18, 22–26].

The 29kDa cytokine-induced protein, CIP29, also known as HCC1 or SARNP, was first identified in a proteomic screen of a hepatocellular carcinoma cell line, although no function was ascribed to the protein [27]. Upregulated in a cytokine-dependent manner and overexpressed in various cancers, CIP29 is a nuclear protein which contains an N-terminal SAP (SAF-A/B, Acinus and PIAS) domain [27, 28]. This structural motif is a DNA binding domain which occurs in a variety of proteins involved in chromosome architecture, DNA repair and RNA processing [29]. Accordingly, CIP29 can interact with DNA and displays an affinity for scaffold attachment region DNA in *in vitro* assays [30]. The yeast orthologue of CIP29, Tho1, was identified as a multicopy suppressor for RNA processing defects of THO complex mutants, implying its involvement in some aspect of RNA processing [31]. Notably, overexpression of Tho1 can suppress mRNA export defects and prevents R-loop formation and the associated gene rearrangements normally seen in THO mutants. Subsequent studies of human CIP29 have variously reported CIP29 as interacting with the TREX mRNA export complex, or as a component of an alternative TREX complex, AREX, further indicating a possible involvement of CIP29 in mRNA export, although the precise nature of its function in RNA processing is still not clear [32, 33]. Given the close association between RNA processing and the cellular response to DNA damage we considered it possible that CIP29 may have a role to play in preventing genome instability. This is particularly the case, given its ability to suppress R-loop-associated phenotypes in yeast. Moreover, physical interactions have been reported between CIP29 and a variety of other RBPs identified as targets of DNA damage-dependent signalling [32].

Here, we have made use of a *X*. *laevis* egg extract system to investigate the possibility that CIP29 is a target of the DNA damage response. Cell-free extracts of *X*. *laevis* eggs represent a tractable system for biochemical analysis, extensively used in the study of cell cycle regulation and DNA damage responses, since they faithfully recapitulate events such as nuclear assembly, DNA replication and activation of DNA damage response signalling [34]. Using this system, we show that CIP29 is rapidly phosphorylated in response to DNA damage as a consequence of PI3KK-dependent DNA damage signalling. We have determined that this damage-dependent phosphorylation of CIP29 relies on ATM activation and we have identified a conserved serine residue in the central region of the CIP29 protein as a damage-dependent phosphorylation site. Finally, we have ruled out a direct role for CIP29 in DNA double-strand break (DSB) repair via DNA end joining in this system.

## Materials and methods

The use of animals (Xenopus laevis) in this work was approved by the Lancaster University Ethical Review Process (ERP) and is covered by Home Office Project Licence 7007392.

### Plasmids and site-directed mutagenesis

The *X*. *laevis* Cip29 clone, IMAGE6868698, was obtained from Source Bioscience and the Cip29b gene (XM_018247919) was synthesised by Eurofins. The complete open reading frames were PCR amplified using TaqPlus DNA polymerase (Agilent) and primers 5’-ACATATGGCGGACACAGTGGAGCTTC and 5’-TTCTCGAGCTAGACAATGCCAAAGCGTTCTGA-TC, containing *Nde*I and *Xho*I restriction sites. The PCR products were cloned into pGEM-T easy (Promega) and pET16b (Novagen) for *in vitro* translation and bacterial expression respectively. Cip29 fragments were PCR amplified using the following primer pairs and cloned into the pGEX-KG expression vector, in-frame with the GST sequence [35]:

F1: 5’-AACATATGGCGGACACAGTGGAGCTTC, 5’-TCTCGAGCTAATTTGCTTCTTCTTCAGCGTG, F2: 5’-AACATATGGAAGAAGCAAATGAAGAAGATGTTTTGG, 5’-TTCTCGAGCTATTTCACCAACTTCTTATC-TGCATTTG, F3: 5’-AACATATGGATAAGAAGTTGGTGAAAATTCC, 5’-TTCTCGAGCTAGGTTGAAATG-CCAAACCGGATG), F4: (5’-AACATATGCGGTTTGGCATTTCAAC, 5’-TTCTCGAGCTATTCCTTCCT-TTTCTTTAGC), F5: (5’-AACATATGCTAAAGAAAAGGAAGGAACGTTTTG, 5’-TTCTCGAGCTAGAC-AATGCCAAAGCGTTCTGATC).

Mutation of potential Cip29 phosphorylation sites was carried out using the QuikChange™ II XL Site-Directed Mutagenesis Kit (Agilent). All constructs were verified by sequencing.

### Recombinant proteins and antibodies

*In vitro* transcription/translation of Cip29 in pGem-T easy was performed using a TNT T7 Quick Coupled Transcription/Translation System (Promega) according to the manufacturer’s instructions. Expression of full length recombinant His_10_-tagged Cip29 protein was induced in *E*.*coli* BL21 (DE3) pLysS (Novagen) by addition of 1mM IPTG for 3h at 37°C. The protein was purified on Nickel-NTA agarose (Qiagen), under denaturing conditions (8M urea), according to the manufacturer's instructions and used for the production of polyclonal antisera (Eurogentec Ltd). α-Cip29 antibodies were affinity purified against the His_10_-Cip29 antigen coupled to Amino-link plus resin (Perbio) according to the manufacturer’s instructions. For phosphorylation experiments, full length recombinant His_10_-tagged Cip29 protein was purified under native conditions, eluted with 20mM MOPS, 500mM NaCl, 0.5M imidazole, then dialysed against 10mM Hepes, pH 7.4, 100mM KCl, 50mM sucrose, 1mM EDTA prior to storage at -80°C. Expression of Cip29 fragments as GST fusions was induced in *E*.*coli* BL21 (Novagen) using 50μM IPTG for 3h at 37°C. GST fusion proteins were purified on glutathione sepharose (GE Healthcare) and eluted with 10mM glutathione. prior to dialysis against 10mM Hepes, pH 7.4, 100mM KCl, 50mM sucrose, 1mM EDTA and storage at -80°C.

Antibodies to *X*. *laevis* Chk1, Chk2 (Cds1), Mre11 and Uhrf1 have been described previously [36, 37, 38]. Phospho-specific antibodies to human Chk1-S345 were purchased from Cell Signaling Technology, anti-His antibody was from Clontech, while HRP-conjugated anti-rabbit and anti-mouse secondary antibodies were from Dako.

### *X*. *laevis* egg extracts and immunodepletion

*X*. *laevis* egg extract was prepared according to the method of Felix *et al* (1989) with some minor modifications as previously described [36, 39]. Activation of the DNA damage response was achieved by addition of a 70mer poly-AT oligonucleotide (AT_70_) to a final concentration of 50ng/μl in the presence of 3mM tautomycin (Calbiochem) as described [40]. Immunodepletion of egg extract was achieved using antibodies crosslinked to protein A sepharose (GE-Healthcare) with dimethyl pimilimidate by the method of Harlow and Lane [41]. For immunodepletion, egg extract was incubated with 40% (v/v) protein A sepharose crosslinked to affinity purified antibodies (α -Cip29, α-Chk1, α-Chk2, α-Mre11) for 45 min at 4°C, with occasional resuspension of the beads. Two rounds of depletion were routinely conducted. Mock-depleted extracts were treated identically except that non-specific rabbit IgGs (Sigma), cross-linked to protein A sepharose, were used for depletion. Kinase inhibitors NU7441 and KU55933 were supplied by Tocris Bioscience and VE821 was from Calbiochem.

### SDS-PAGE and immunoblotting

Protein samples were run on 12% SDS-PAGE gels and proteins visualised by staining with either Coomassie Brilliant Blue or with SYPRO Ruby protein gel stain (Invitrogen) according to the manufacturer’s basic protocol. Broad Range Pre-stained Protein Marker (NEB) was routinely included. For immunodetection, proteins were transferred to a nitrocellulose membrane (Nitrobind, Osmonics Ltd) by semi-dry transfer. Membranes were blocked (5% non-fat milk powder, 0.1% Tween 20 in PBS) and incubated with primary antibodies overnight at 4°C. Membranes were then incubated with HRP-conjugated anti-rabbit secondary antibody and bands visualised using enhanced chemiluminescence.

For detection of Cip29 phosphorylation, protein samples were run on 8% SDS-PAGE gels containing 30μM MnCl_2_ and 15μM Phos-tag (Wako Pure Chemical Industries Ltd). Size markers were not routinely included on Phos-tag gels, in line with the manufacturer’s recommendations. Since Phos-tag gel separation of phosphoprotein isoforms is very sensitive to levels of protein loading, we routinely ran 10μg protein per lane, for egg extract samples, while immunoprecipitated samples were supplemented with 2μg of Cip29-depleted egg extract to optimise band separation. Following electrophoresis, Phos-tag-acrylamide gels were soaked in wet transfer buffer (48mM Tris-base, 39mM glycine 20% methanol) containing 1mM EDTA for 15 minutes. Gels were then soaked in wet transfer buffer without 1mM EDTA before protein transfer to nitrocellulose using a Mini-Trans Blot cell system (BioRad).

### Phosphorylation of recombinant proteins in *X*. *laevis* egg extract

To determine phosphate incorporation, 5μg of recombinant GST-Cip29 or His_10_-Cip29 protein was added to 30μl of *X*. *laevis* egg extract supplemented with 3μM tautomycin and 1μCi of [γ-32P] ATP, in the presence or absence of 50ng/μl AT_70_. Samples were incubated at 21°C for 1 hour. For recovery of GST-tagged proteins, samples were diluted with 2 volumes of GST buffer (50mM Tris-HCl, pH 7.5, 250mM NaCl, 0.15 Triton X-100), applied to 20μl glutathione sepharose and mixed for 1h at 4°C. The GST-Cip29-bound beads were then washed with GST buffer and the bound proteins eluted with 30μl GST elution buffer (50mM Tris.HCl pH 8, 15mM reduced glutathione). For recovery of His_10_-tagged proteins, samples were diluted with 2 volumes of His bind buffer (20mM MOPS, 500mM NaCl, 5mM imidazole), applied to 20μl nickel NTA agarose (Qiagen) and mixed for 1h at 4°C. The His_10_Cip29-bound beads were washed with wash buffer (20mM MOPS, 500mM NaCl, 60mM imidazole) and the bound proteins eluted with 30μl elution buffer (20mM MOPS, 500mM NaCl, 1M imidazole). Eluted proteins were resolved by SDS-PAGE, visualised by SYPRO Ruby staining, and protein levels quantified using ImageQuant TL software (version 2003.02, GE Healthcare). SDS-PAGE gels were then dried and exposed to a phosphorimager screen and ^32^P-incorporation was quantified using the ImageQuant TL software. SYPRO Ruby detection and phosphorimaging were performed on a Typhoon 9410 flatbed scanner (Amersham Biosciences). ^32^P-incorporation for each GST-Cip29 fragment was normalised for protein recovery.

For Phos-tag gel analysis, 3.5μg of recombinant His_10_-Cip29 proteins were added to 20μl of *X*. *laevis* egg extract supplemented with 3μM tautomycin, in the presence or absence of 50ng/μl AT_70_. Samples were incubated at 21°C for 1 hour before recovery of His_10_-tagged proteins as described above.

### DNA end joining assay

Linear DNA repair substrate with ^32^P-labelled 3’ hydroxyl overhang ends was produced as previously described [42]. DNA end joining reactions were performed essentially as described in Taylor *et al* [36]. Briefly, 20 μl of egg extract was combined with 12.5ng of linear DNA repair substrate and 1 μl of NHEJ mix (1mM ATP, 1mM MgCl_2_ and 50 μM dNTPs) and incubated at 21°C for 6h. The reaction was stopped by the addition of lysis buffer (0.3M NaCl, 2mM Tris-HCl, pH 6.7, 10mM EDTA, 1% (w/v) SDS and 1 mg/ml proteinase K) and incubation at 65°C for 3 hours. Following phenol/chloroform extraction, the DNA was ethanol precipitated, dissolved in 10mM Tris-HCl containing 1mM EDTA, then digested with 10 units *Bst*XI (NEB) followed by 20 units *Taq*^∝^I (NEB). The DNA was ethanol precipitated and dissolved in loading buffer (20mM EDTA, bromophenol blue, in formamide). Samples were resolved on 20% sequencing gels (SequaGel National Diagnostics) (40W, 3h), then exposed to a phosphorimager screen at -20°C. Repair products were visualised by scanning on a Typhoon 9410 (Amersham Biosciences).

## Results

### Identification of a *Xenopus* orthologue of Cip29

**Fig 1:**
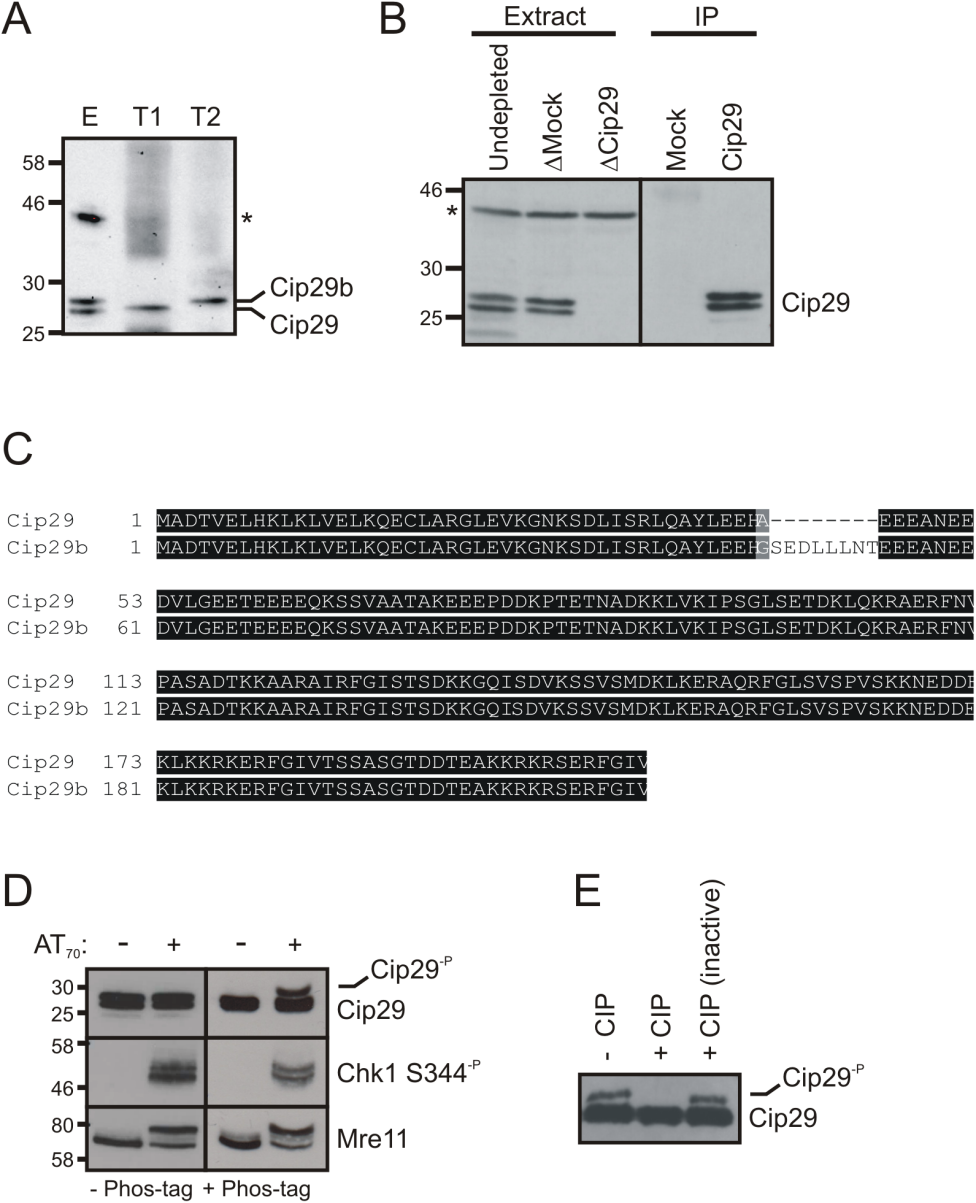
Detection of Cip29 and phosphorylated Cip29 proteins in *X*. *laevis* egg extract. (A) Immunodetection of Cip29 on a Western blot of *X*. *laevis* egg extract (E), *in vitro* translated Cip29 (T1) and *in vitro* translated Cip29b (T2). A non-specific band detected by the α-Cip29 antibodies in *X*. *laevis* egg extract is indicated with an asterisk. Molecular weight markers (kDa) are indicated on the left. (B) Western blot of Cip29 in *X*. *laevis* egg extract, undepleted or immunodepleted with either non-specific rabbit IgGs (mock) or α-Cip29 antibodies (left panel), and in the immunoprecipitated samples (right panel). The non-specific band (*) serves as a loading control for extract samples. (C) Amino acid sequence alignment of *X*. *laevis* Cip29 and Cip29b. (D) Western blots of egg extract incubated with or without 50ng/μl AT_70_ (21^°^C, 1h). Samples were resolved by electrophoresis on a standard 12% SDS-PAGE gel (left panel) and an 8% SDS-PAGE gel containing 15μM Phos-tag (right panel) before immunoblotting with the indicated antibodies. Modified Cip29 protein is denoted by Cip29^-P^ (E) Cip29 was immunoprecipitated from egg extract supplemented with 50ng/μl AT_70_ (21^°^C, 1h). The immunoprecipitation reactions were treated with either 1 x NEBuffer 3 (-CIP), or NEBuffer 3 containing 10 units of CIP, or 10 units of heat-inactivated CIP (21^°^C, 1h) before glycine elution of the immunoprecipitated proteins. Eluted protein was supplemented with 2μg of Cip29-depleted extract before separation on Phos-tag SDS-PAGE, to optimise resolution of the modified form of Cip29.

We first identified the *X*. *laevis* orthologue of Cip29 through database searches using the human CIP29 amino acid (aa) sequence (BC007099). This identified a *X*. *laevis* sequence (BC133773) with significant identity to the human protein. This sequence spans a predicted open reading frame of 208aa that is 72% identical to the human CIP29 protein and contains conserved features such as the N-terminal SAP motif and two C-terminal nuclear localisation sequences. Polyclonal antisera were raised against bacterially-expressed full-length *X*. *laevis* Cip29. Western blotting confirmed that these antibodies recognise a single band of approximately 27kDa corresponding to the *in vitro* translated Cip29 protein while, in *X*. *laevis* egg extract, they recognise two bands of approximately 27 and 28kDa, as well as a non-specific band of approximately 43kDa (Fig 1A). Both bands of the Cip29 doublet, but not the non-specific band, can be immunoprecipitated using α-Cip29 antibodies (Fig 1B) suggesting that both bands of the doublet represent forms of *X*. *laevis* Cip29. Further database searching identified an alternative transcript of Cip29 (XM_018247919), which we have termed Cip29b. This alternative transcript arises from read-through of the splice site at the end of exon 2 resulting in an 8aa insertion in the expressed protein (Fig 1C). This alternatively expressed form likely accounts for the larger of the two Cip29 bands observed in *X*. *laevis* egg extracts.

Next, we examined Cip29 mobility on SDS-PAGE under conditions where the DNA damage response is activated in *X*. *laevis* egg extract. When the DNA damage response is triggered by addition of the double-stranded oligonucleotide AT_70_, the checkpoint protein, Chk1, and the DNA DSB repair protein, Mre11, are both activated by phosphorylation (Fig 1D). Chk1 phosphorylation is demonstrated using a phospho-specific antibody against the activating phosphorylation on Ser344, while Mre11 phosphorylation is discernible by the appearance of a lower mobility form of the protein on SDS-PAGE. In contrast, no detectable mobility shift was seen for Cip29, in response to AT_70_, when analysed by normal SDS-PAGE. Nevertheless, many protein phosphorylation events do not result in a detectable change in apparent molecular weight when analysed in this way. Therefore, we decided to use a modified electrophoretic separation method to detect phosphoprotein isoforms. Inclusion of the Phos-tag ligand, which binds to and selectively decreases the mobility of phosphorylated proteins, can significantly increase the level of separation between hyper- and hypo-phosphorylated forms [43]. Using a Phos-tag gel, we were able to identify a slower migrating form of Cip29, distinct from the main Cip29 protein doublet, in AT_70_-treated but not untreated extracts. The fact that this slower migrating form was only apparent in the presence of the phosphate affinity ligand is strongly suggestive that it represents a phosphorylated form of Cip29 (Fig 1D). To further confirm this, we immunoprecipitated Cip29 from AT_70_-treated egg extract and then treated the immunoprecipitates with calf intestinal alkaline phosphatase (Fig 1E). Phosphatase treatment clearly abolishes the slower migrating form of Cip29, while not affecting the migration of the main Cip29 doublet, confirming the upper band as a phosphorylated isoform.

### Cip29 phosphorylation is induced by DNA damage in a checkpoint-dependent manner

**Fig 2:**
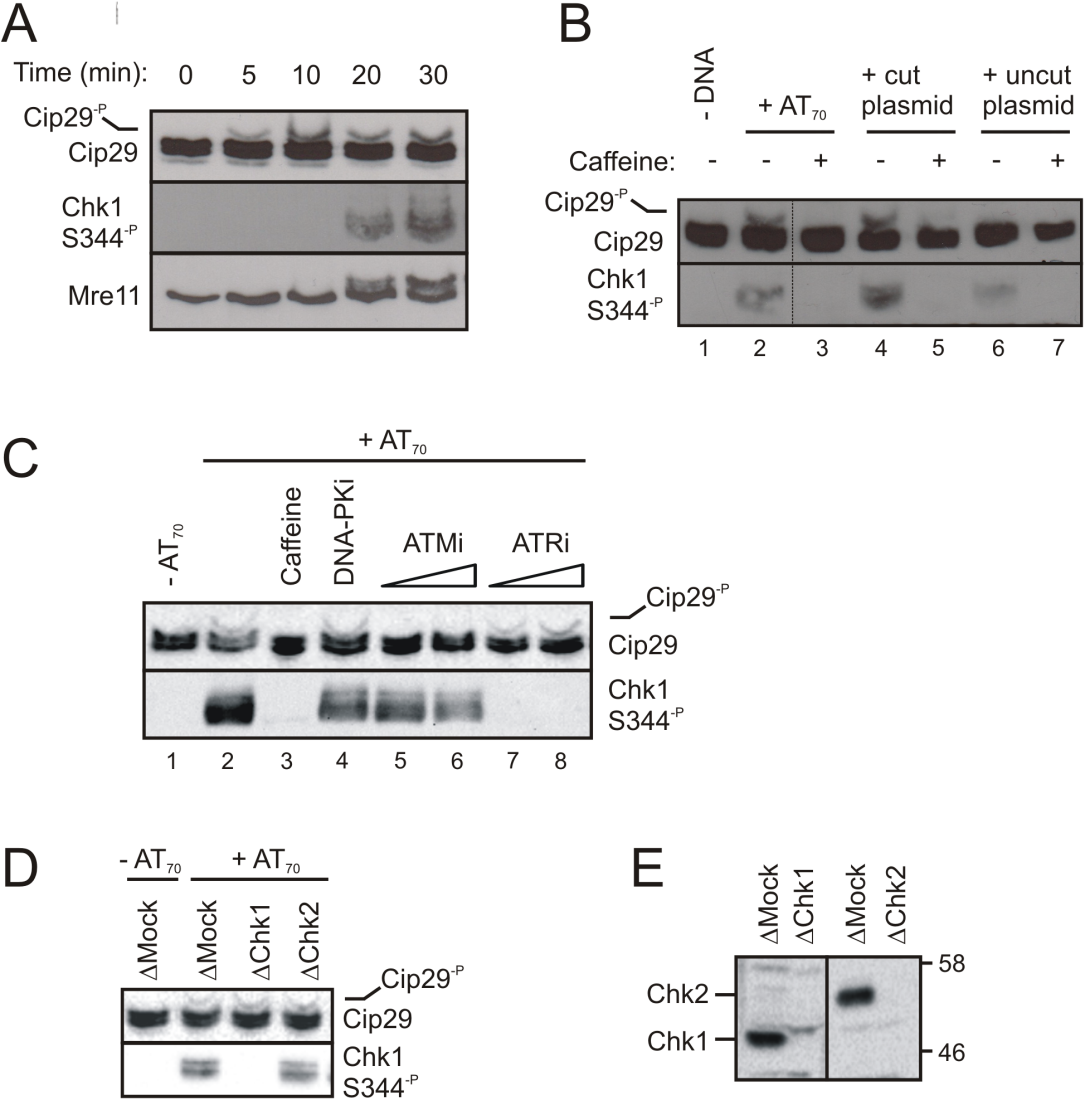
Cip29 is phosphorylated in a DNA damage- and ATM-dependent manner in *X*. *laevis* egg extract. (A) Egg extract was supplemented with 50ng/μl AT_70_ and incubated at 21^°^C for the indicated times before reactions were stopped by addition of 5 x SDS-PAGE gel loading buffer. Samples were analysed by Phos-tag SDS-PAGE and western blotting with the indicated antibodies. (B) Egg extract was incubated with either AT_70,_ cut plasmid or uncut plasmid (50ng/μl), in the absence or presence of caffeine (5mM) (21^°^C, 1h) before analysis by Phos-tag SDS-PAGE and western blotting. Dotted line indicates removal of intervening lanes on the same gel for the sake of clarity. (C) Egg extract was incubated in the absence or presence of 50ng/μl AT_70_ supplemented with 5mm caffeine, 10μM DNA-PKi (NU7441), 20μM and 40μM ATMi (KU55933) or 20μM and 40μM ATRi (VE821) as indicated (21^°^C, 1h), and samples were analysed by Phos-tag SDS-PAGE and western blotting. (D) Egg extract, immunodepleted with non-specific rabbit IgGs (mock), α-Chk1 or α-Chk2 antibodies, was incubated with 50ng/μl AT_70_ (21^°^C, 30min) and analysed by Phos-tag SDS-PAGE and western blotting. (E) Western blot of immunodepleted extracts from (D) indicating depletion efficiency for Chk1 and Chk2. Molecular weight markers (kDa) are indicated on the right.

We performed a timecourse to examine the kinetics of Cip29 phosphorylation in AT_70_-treated egg extract (Fig 2A). This analysis showed that Cip29 is phosphorylated rapidly, within 5min of AT_70_ addition to the extract. In contrast, phosphorylation of the DNA damage response factors, Chk1 and Mre11, can only detected from 20min in this experimental system. These data reveal Cip29 phosphorylation to be an early consequence of activation of the DNA damage response in *X*. *laevis* egg extracts.

AT_70_ is thought to generate several structures including double-stranded DNA ends, primed single-strand DNA ends and replication fork mimics, and serves to activate both ATM- and ATR-dependent DNA damage response pathways. In order to elucidate the trigger for Cip29 modification in more detail we first of all compared AT_70_ with plasmid DNA, which was either uncut or linearised by restriction enzyme digestion (Fig 2B). The cut plasmid mimics DNA damage in the form of DNA DSBs, while uncut plasmid served as a control to ascertain whether the presence of DNA was in itself sufficient to trigger Cip29 phosphorylation. Both the AT_70_ and the linearised plasmid effectively activated the DNA damage response, as evidenced by Chk1 Ser344 phosphorylation, and both induced phosphorylation of Cip29 (Fig 2B, lanes 2 and 4). In contrast, addition of closed circular uncut plasmid did not cause Cip29 phosphorylation (Fig 2B, lane 6), indicating that it is the dsDNA ends, rather than the presence of DNA alone, that serves as the trigger for this post-translational modification. Somewhat surprisingly, some Chk1 Ser344 phosphorylation was detected in the sample containing uncut plasmid, albeit at a reduced level compared with either of the DNA damage mimics. It is likely that this is due to the presence of a small amount of nicked DNA in the plasmid preparation or induced by nucleases following its addition to the *X*. *laevis* extract.

To ascertain whether Cip29 phosphorylation occurred as a result of DNA damage signalling through one of the PI3K-related kinases, ATM, ATR or DNA-PK, we examined the effect of caffeine, a potent inhibitor of PI3KK activity. Our data show that caffeine effectively abolished both Cip29 and Chk1 phosphorylation in the presence of DNA damage (Fig 2B, lanes 3 and 5), confirming that DNA damage-dependent phosphorylation of Cip29 requires the activity of at least one of the DNA damage-activated kinases, ATM, ATR or DNA-PK. To determine which of these kinases was important for Cip29 phosphorylation we tested the effect of specific inhibitors for each of the three PI3KKs (Fig 2C). This analysis showed that both DNA-PK and ATR activities are dispensable for Cip29 modification (Fig 2C, lanes 4, 7 and 8), but that ATM kinase activity is absolutely required for AT_70_-dependent phosphorylation of Cip29 (Fig 2C, lanes 5 and 6). However, DDR-dependent phosphorylation of Cip29 does not depend on the activity of either of the downstream checkpoint kinases, Chk1 or Chk2, since depletion of either of these proteins has no noticeable effect on AT_70_-induced Cip29 modification (Fig 2D and 2E).

### DNA damage response-dependent Cip29 phosphorylation occurs at a conserved serine residue

**Fig 3:**
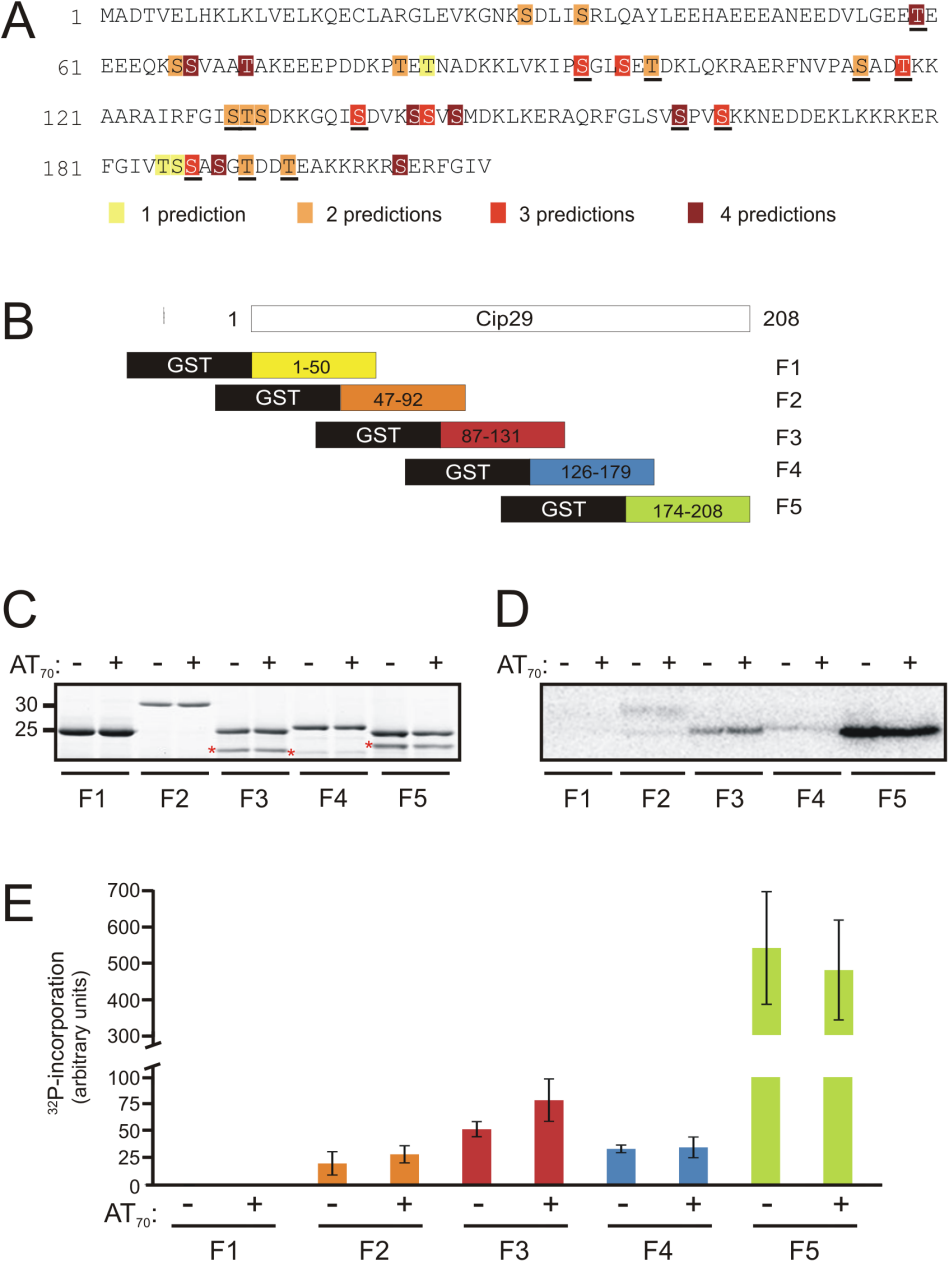
Phosphorylation of GST-Cip29 protein fragments in *X*. *laevis* egg extract. (A) *X*. *laevis* Cip29 amino acid sequence indicating potential phosphorylation sites as predicted using the protein phosphorylation prediction tools, NetPhos 2.0, PHOSIDA, KinasePhos 2.0 and Scansite. The number of times each residue is predicted as a candidate phospho-site is indicated by shading, as described in the key. Ser/Thr residues that are conserved between *X*. *laevis* Cip29 and other vertebrates are underlined. (B) Schematic representation of GST-Cip29 fragments. Five Cip29 protein fragments (F1-F5), encompassing the entire Cip29 protein sequence, were each expressed with an N-terminal GST tag to facilitate purification. (C) Purified GST-Cip29 fusion proteins, F1-F5, (5μg each) were incubated in egg extract supplemented with ^32^P-ATP, in the absence or presence of 50ng/μl AT_70,_ (21^°^C, 1h), then the recombinant proteins were recovered and subject to SDS-PAGE. Proteins were visualised by Sypro Ruby staining. Premature termination products or degradation products are denoted with asterisks. (D) After staining and protein quantification, the SDS-PAGE gel from (C) was dried and exposed to a phosphorimager screen for 48h to determine ^32^P-incorporation. (E) ^32^P-incorporation is presented for each fragment, in the absence or presence of AT_70_, relative to the amount of protein per band. The data shown in the graphs correspond to the mean of three independent experiments, and error bars indicate the SD. Note the axis break and scale change on the *y* axis to accommodate the high incorporation level in F5, relative to F1-F4.

As a first step towards identifying the site of the DDR-dependent phosphorylation on Cip29, we analysed the entire Cip29 sequence for potential phosphorylation sites using four different phospho-site prediction tools; NetPhos 2.0, PHOSIDA, KinasePhos 2.0 and Scansite (Fig 3A) [44–47]. This analysis revealed a number of potential phosphorylation sites distributed throughout the protein, fifteen of which were identified by at least three of the prediction programmes. Of these, seven (Thr59, Ser95, Thr118, Ser139, Ser162, Ser165 and Ser187) are conserved in other vertebrates. Notably, no canonical PI3KK consensus phosphorylation sites (SQ or TQ) are present in Cip29.

**Fig 4:**
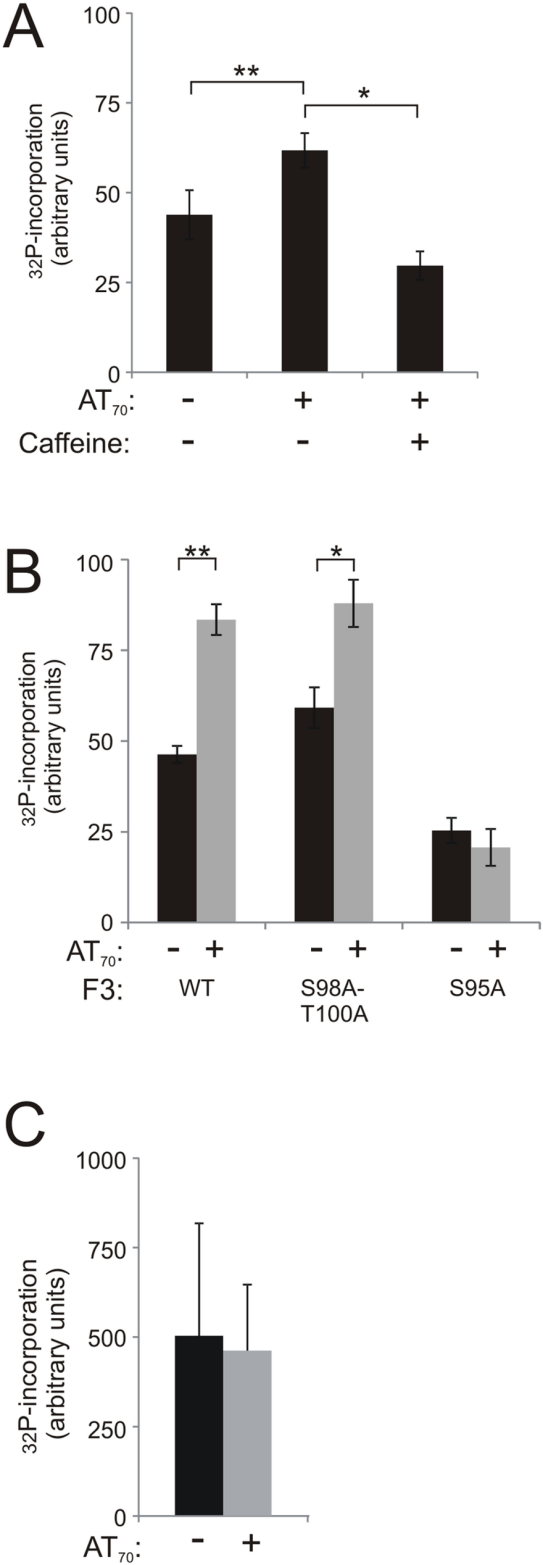
Damage-dependent phosphorylation of Cip29 occurs on a conserved Ser residue of fragment 3. (A) F3 (5μg) was incubated in egg extract with ^32^P-ATP in the absence or presence of 50ng/μl AT_70_ and 5mM caffeine (21^°^C, 1h). F3 was recovered from the extract and ^32^P-incorporation was determined for each condition. (B) 5μg each of F3 (WT), F3 containing the double mutation S98A/T100A or the single mutation S95, were incubated as in (A) and ^32^P-incorporation determined for each fragment. (C) 5μg of full length His_10_-Cip29 was incubated as in (A) and ^32^P-incorporation was determined. In each case, the data represent an average of three independent experiments where error bars indicate the SD. Significance of the observed differences was evaluated using Student’s *t* test (*P 0.01–0.05; **P 0.001–0.01).

We decided, therefore, to refine our search for the phosphorylation target site by monitoring the incorporation of radiolabelled phosphate into a series of recombinant Cip29 fragments. We generated a series of five Cip29 fragments (F1-F5) as glutathione S-transferase (GST) fusion proteins, corresponding to amino acids 1–50 (F1), 47–92 (F2), 87–131 (F3), 126–179 (F4) and 174–208 (F5) of the Cip29 protein (Fig 3B). The purified recombinant proteins can be seen in Fig 3C. All appear to be of the expected size on SDS-PAGE with the exception of fragment 2, which runs with a significantly higher apparent molecular weight, perhaps due to the extensively acidic nature of this particular Cip29 fragment (approximately 40% acidic residues). Each of these fusion proteins was incubated in *X*. *laevis* egg extract supplemented with ^32^P-ATP, in both the absence and presence of AT_70_, and the amount of radiolabelled phosphate incorporated into each fragment was determined (Fig 3D and 3E). From this analysis we could not detect any appreciable phosphorylation of fragment 1 consistent with the relatively weak phospho-site prediction for this fragment. Fragment 2 and fragment 4 each contain a number of strongly predicted potential phosphorylation sites and both fragments did indeed exhibit a degree of phosphate incorporation. However, there was little change in the extent of this incorporation following the addition of AT_70_, implying constitutive rather than damage-inducible phosphorylation of fragments 2 and 4. Fragment 5 was extensively phosphorylated but, once again, we saw no increase in phosphorylation of this region of Cip29 after activation of the DNA damage response in egg extract. In contrast, fragment 3, although phosphorylated to a lesser extent than fragment 5, was the only protein to show a reproducible increase in labelled phosphate incorporation in the presence of AT_70_, suggesting that a damage-dependent phosphorylation site resides within this central region of the Cip29 protein. Moreover, this DNA damage-dependent phosphorylation of fragment 3 is sensitive to inhibition by the general PI3KK inhibitor caffeine, confirming that it is dependent on activation of the DNA damage checkpoint (Fig 4A).

Fragment 3 contains seven potential phosphorylation targets, namely Ser95, Ser98, Thr100, Ser115, Thr118, Ser130 and Thr131. Of these, Ser130 and Thr131, at the extreme C-terminus of fragment 3, are also present in fragment 4 which does not exhibit any discernible AT_70_-dependent phosphorylation, and so we considered them unlikely candidates for DDR-dependent phosphorylation. To determine the contribution of the remaining serine/threonine sites, we made a series of mutations in fragment 3 in which various serine or threonine residues were substituted with non-phosphorylable alanine, and we examined the effects of these mutations on damage-dependent ^32^P-incorporation. Mutation of Ser98 and Thr100 had no effect on the AT_70_-dependent phosphorylation of fragment 3. In contrast, mutation of Ser95 to alanine completely abolished AT_70_-dependent phosphorylation of fragment 3 (Fig 4B).

**Fig 5:**
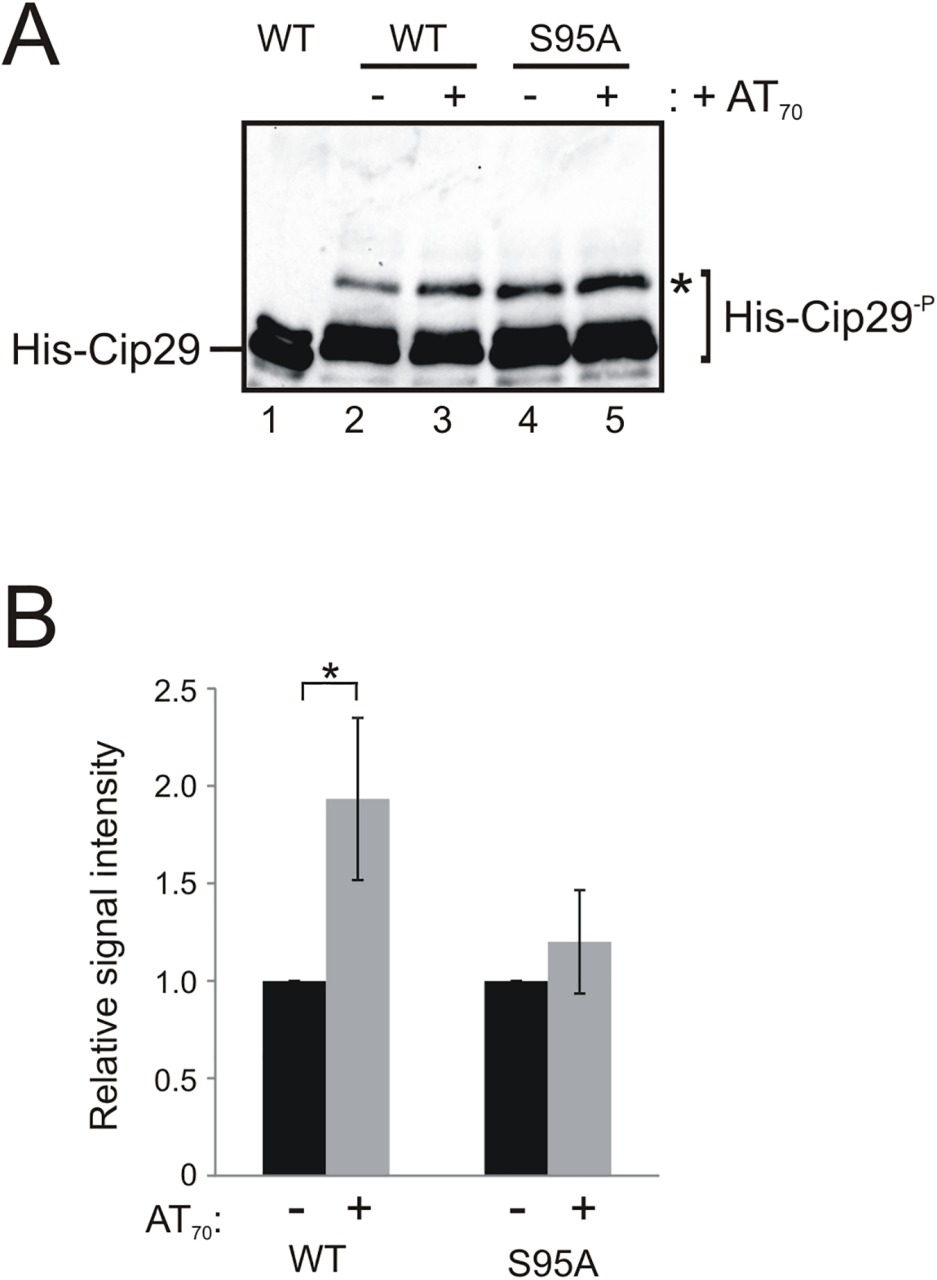
Damage-dependent phosphorylation of recombinant full length Cip29. (A) Western blot of full length His_10_-Cip29 (WT) (lane 1) alongside His_10_-Cip29 (WT and S95A) incubated in egg extract with or without 50ng/μl AT_70_ (21^°^C, 1h) and recovered on nickel NTA agarose. Samples were resolved by electrophoresis on an 8% SDS-PAGE gel containing 15μM Phos-tag before immunoblotting with the anti-His antibodies. A modified Cip29 isoform is denoted by *. (B) Quantification of signal intensity of the modified Cip29 isoform (*) relative to the total Cip29 signal in each lane, expressed as fold-increase for AT_70_-treated extract relative to untreated extract. The data represent an average of three independent experiments where error bars indicate the SD. Significance of the observed differences was evaluated using Student’s *t* test (*P 0.01–0.05).

To further confirm the importance of Ser95 for the DDR-dependent phosphorylation of Cip29, we extended our analysis to examine this phosphorylation in the context of the complete Cip29 protein. Phosphate incorporation into full length His_10_-tagged Cip29 was analysed in *X*. *laevis* egg extract, in both the absence and presence of AT_70_, but perhaps unsurprisingly given the degree of phosphorylation that we noted in our fragment analysis, extensive constitutive phosphorylation of the full length Cip29 protein masked any evidence of DNA damage-dependent modification (Fig 4C). We therefore decided to examine the phosphorylation-dependent mobility shift of the full length protein by Phos-tag gel analysis. Full length His_10_-tagged Cip29 was incubated in *X*. *laevis* egg extract, in both the absence and presence of AT_70_, recovered on nickel NTA agarose, then analysed by Phos-tag SDS-PAGE (Fig 5A). This analysis indicates that the recombinant protein is extensively phosphorylated upon incubation in *X*. *laevis* egg extract, exhibiting lower mobility than recombinant protein not incubated in extract (Fig 5A, lane 1) and revealing the appearance of a further modified form of the protein (*), regardless of DDR activation (Fig 5A, lanes 2–5). Under the conditions tested, it was not possible to resolve a DNA damage-dependent isoform away from this constitutively modified band. However, quantitation of the Western blot data revealed that the relative signal intensity of this modified form is significantly increased following DNA damage (Fig 5B). Moreover, this damage-dependent increase is largely abolished by mutation of Ser95. Taken together, these data suggest that the conserved Ser95 residue is a major site of ATM-dependent phosphorylation of Cip29 following activation of the DNA damage response in *X*. *laevis* egg extract.

### Cip29 is not required for efficient DNA end joining in *X*. *laevis* egg extract

**Fig 6:**
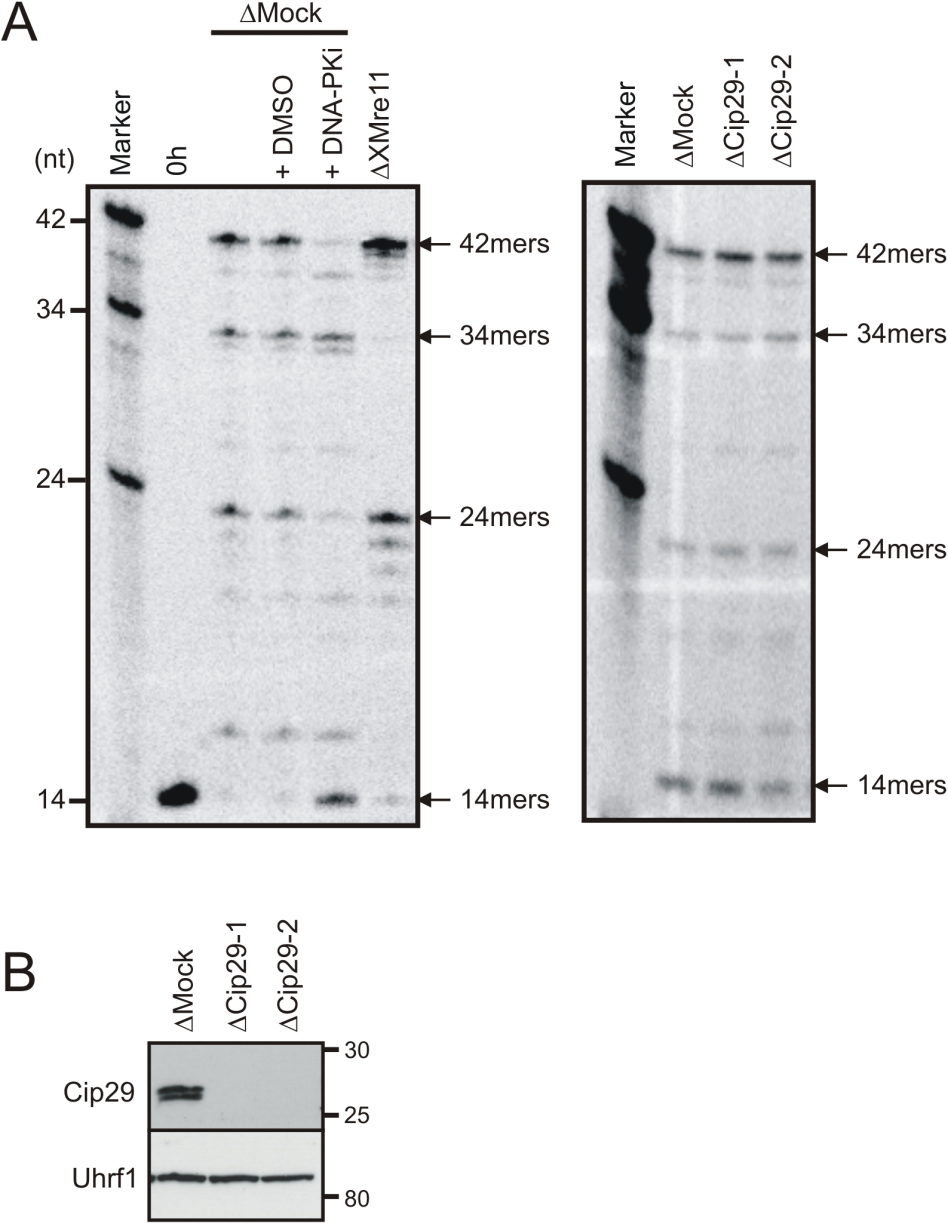
Analysis of DNA end joining in Cip29-depleted egg extract. (A) Sequencing gel analysis of DNA end joining in *X*. *laevis* egg extracts using a defined repair substrate. Linearized repair substrate was incubated at 1ng/μl in mock-depleted, Mre11-depleted or Cip29-depleted extract (21°C, 6h). Where indicated, DMSO was added to a final concentration of 0.4% and DNA-PKi (NU7441), dissolved in DMSO, was added to a final concentration of 8μM. Cip29-depletion was performed with two different α-Cip29 antibodies (ΔCip29-1 and ΔCip29-2). Following incubation, plasmid DNA was recovered, digested with Taq^α^I and BstXI, resolved on a 20% denaturing polyacrylamide gel and exposed to a phosphorimager screen. Linearized substrate added to mock-depleted extract and processed immediately serves as an unrepaired control (0h). (B) Western blot of immunodepleted extracts from (A) indicating the depletion efficiency for Cip29 with both α-Cip29 antibodies. Uhrf1 serves as a loading control. Molecular weight markers (kDa) are indicated on the right.

We have shown that Cip29 is phosphorylated in response to dsDNA ends in a checkpoint-dependent manner, and that this post-translational modification occurs rapidly after DNA damage treatment, even preceding that of the known ATM target, the DNA repair factor Mre11. Therefore, we reasoned that phosphorylation of Cip29 could be an important element in the cellular response to DNA damage which may, for example, influence the repair of DNA DSBs. *X*. *laevis* egg extracts possess very effective DNA end joining activities for the repair of DNA DSBs. These include a highly accurate classical non-homologous end joining pathway, dependent on DNA-PK, as well as an error-prone microhomology-mediated end joining mechanism dependent on the Mre11-Rad50-Nbs1 complex [36, 48–49]. To investigate a potential role for Cip29 in DSB repair we employed an end joining assay which allows for analysis of DSB repair products at the nucleotide level, and so differentiates between accurate, DNA-PK-dependent repair and resection-associated, Mre11-dependent repair [36]. This assay uses an internally radiolabelled linear plasmid as the repair substrate. After appropriate enzymatic digestion of the repair substrate and resolution of the digested products on a sequencing gel, this unrepaired DNA template can be detected as a 14mer (Fig 6A, left panel). Incubation of the repair substrate in *X*. *laevis* egg extract leads to the formation of a variety of repair products which can be detected in this assay as follows; 42 nucleotide (nt) oligomers resulting from accurate, DNA-PK-dependent head-to-tail end joining of the linear plasmid DNA, 24nt DNA-PK-dependent head-to-head end joining products and, at 34-39nt (predominantly 34nt), inaccurately repaired products in which Mre11-dependent head-to-tail end joining is accompanied by the resection of several nucleotides. As shown in Fig 5A (left panel), treatment of *X*.*laevis* egg extract with a DNA-PK inhibitor (NU7441) prevents formation of the 42 and 24mer accurate end joining products, while immunodepletion of Mre11 abolishes the 34mer resection-dependent repair product. Immunodepletion of Cip29, using either of two different α-Cip29 antibodies, did not affect the level of either the accurate or resection-mediated repair products, relative to mock-depleted extract, in this assay (Fig 6A, right panel). The efficiency of Cip29 immunodepletion by either antibody was confirmed by Western blotting (Fig 5B). These data indicate that Cip29 is not required for the effective function of either of these DNA DSB repair mechanisms in *X*. *laevis* egg extracts.

## Discussion

RNA processing is increasingly being recognised as an important element in the maintenance of genome integrity since it helps both to prevent genetic damage, through the avoidance of cotranscriptional R-loop formation, and to promote effective DNA damage responses, through regulated expression of various DDR proteins [5, 6]. Moreover, a growing list of RNA processing factors has been identified that are either recruited to sites of DNA damage, or which interact with known DNA repair factors, suggesting that they may play a direct role in DNA damage detection, signalling and/or repair [19–21, 50–52]. Consistent with the involvement of RNA processing factors in diverse aspects of the DNA damage response, a number of RBPs have been identified as targets of DNA damage-dependent signalling.

Previous studies have identified CIP29 as a putative RNA export factor [32, 33].We note that in one of these studies, CIP29 was reported to interact with a variety of other RNA processing factors, including several, such as FUS, DDX1, Matrin 3 and the SAP domain-containing protein, hnRNPU, which are known to undergo DNA damage-dependent phosphorylation [53–57]. Despite this, DNA damage-dependent phosphorylation of CIP29 itself has not previously been reported. In the current study however, we have demonstrated that Cip29 is similarly subject to DNA damage-dependent phosphorylation, in *X*. *laevis* egg extracts. We have shown that Cip29 is rapidly phosphorylated in response to dsDNA ends and that caffeine-dependent inhibition of DNA damage signalling prevents this post-translational modification. These data reveal the damage-dependent phosphorylation of Cip29 to be an early event in the DDR but one that occurs downstream of PI3KK-dependent DNA damage signalling. Through the use of specific PI3KK inhibitors we were able to ascertain that DNA damage-dependent phosphorylation of Cip29 is not dependent on either ATR or DNA-PK, but is a consequence of ATM kinase activity. However, this phosphorylation event does not appear to rely on the major downstream kinase of the ATM signalling pathway, Chk2, since depletion of Chk2 has no appreciable effect on Cip29 phosphorylation after damage. Instead, our data indicate that Cip29 is either directly targeted by ATM after DNA damage or is subject to phosphorylation by another, as yet unknown kinase, activated by the ATM signalling pathway. Several candidate kinases acting downstream of ATM could potentially account for Cip29 phosphorylation, including, for example, cyclin-dependent kinase 5, Polo-like kinase1 or the mitogen-activated protein kinase family member, c-Jun kinase, although the rapidity of the Cip29 phosphorylation response, which precedes even that of the known ATM target Mre11, may point towards direct targeting by ATM [58].

The serine-glutamine (SQ) or threonine-glutamine (TQ) sequence motif has been defined as the canonical ATM consensus phosphorylation site, while the presence of hydrophobic or negatively-charged residues around the S/TQ sequence seems to further favour ATM-dependent phosphorylation [59, 60]. Our analysis of potential phosphorylation target sites in Cip29 did not reveal the presence of any canonical S/TQ ATM consensus sites. However, through an *in vitro* labelling assay, we were able to identify the conserved serine residue, Ser95, as the DNA damage-dependent phosphoacceptor site in the central region of Cip29. Although Ser95 is not succeeded by glutamine it is surrounded by hydrophobic residues; isoleucine and proline at positions N-2 and N-1 respectively and glycine and leucine at positions N+1 and N+2, followed by a polar serine residue at N+3 (IP**S**GLS). It may be that this sequence context is sufficient to promote ATM phosphorylation of Cip29 Ser95. ATM phosphorylation of a target serine lacking a glutamine at position N+1 is clearly possible, since the centrosomal protein, Cep63, is known to undergo DNA damage-dependent phosphorylation by ATM at a serine residue within the GS**S**LES sequence, in which the serine phosphoacceptor is flanked by hydrophobic (G, L), negatively-charged (E) and polar (S) residues [61]. Certainly, Ser95 is not very strongly predicted as a phospho-target site of any other Ser/Thr kinases, which may operate downstream of ATM, yet whether the ATM-dependent modification of Cip29 at Ser95 in response to DNA damage occurs directly or indirectly remains to be determined. Moreover, we cannot fully exclude the possibility that further Cip29 residues may additionally serve as DDR-dependent phosphoacceptor sites although, if present, they have proved refractory to our current analysis, given the extensive constitutive phosphorylation of Cip29 in *X*. *laevis* egg extracts. It is possible that the extent of this constitutive phosphorylation may also have proved a confounding factor for proteomic approaches, which have previously failed to reveal DNA damage-dependent phosphorylation of CIP29 [62, 63]. Phosphorylation of RNA processing factors in response to DNA damage signalling may alter their activity, their subcellular localisation and their interactions with other proteins or with their target mRNAs. This may have a variety of consequences, ranging from gene-specific downregulation or upregulation of expression, recruitment to, or exclusion from, sites of transcription or DNA damage, and regulated association with other factors such as DNA repair proteins. Evidence from both yeast and human cells suggests that CIP29 is required for efficient mRNA export and gene expression [31, 33]. These activities may conceivably be affected, either positively or negatively, by ATM-dependent phosphorylation after DNA damage. In this way, CIP29 may play a role in the global repression of gene expression after DNA damage or in the specific stabilisation and expression of DDR-associated genes in these circumstances. In addition, since CIP29 has been reported to interact with a variety of factors that are either recruited to DNA damage sites and/or interact with DSB repair proteins, we considered the possibility that CIP29 may influence DNA repair rather more directly than at the level of gene expression. If so, it could be this aspect of CIP29 activity which is the regulatory target of ATM-dependent damage signalling. *X*. *laevis* egg extracts exhibit efficient DNA end joining activity, the predominant mechanism for repair of DNA DSBs in this experimental system. Therefore, we used a plasmid-based DNA end joining assay to determine whether Cip29 functions in this aspect of DNA DSB repair in *X*. *laevis* egg extracts. Our analysis demonstrated that Cip29 is not required for effective DNA end joining in *X*. *laevis* egg extracts. On this basis, it seems that the ATM-dependent phosphorylation of Cip29 which we have reported here, may well relate to a role in the regulation of Cip29-dependent gene expression after DNA damage, rather than a direct role in DSB repair. However, we cannot entirely rule out a possible role for Cip29 in homologous recombination repair and this remains the subject of future investigations.

## Conclusions

Our data demonstrate that *X*. *laevis* Cip29, a putative RNA export factor, is rapidly phosphorylated in response to DNA double-strand breaks in *Xenopus* egg extracts. This DNA damage-inducible phosphorylation is dependent on the ATM checkpoint kinase and we have identified the damage-dependent phosphorylation site as a conserved serine residue, Ser95. We have determined that Cip29 is not required for efficient DNA end joining repair in *X*. *laevis* egg extracts and suggest that the damage-dependent modification of Cip29 may instead relate to a role in regulation of gene expression after DNA damage.
